# Supplementary material for: Analysis of PPARγ Signaling Activity in Psoriasis
Source: Int J Mol Sci. 2021 Aug 10;22(16):8603. doi: 10.3390/ijms22168603 (PMC8395241; doi:10.3390/ijms22168603)
Supplement: Supplementary file 1 [file ijms-22-08603-s001.zip › Supplemental materials_Analysis of PPARg signaling activity in psoriasis/Pathway models/Models images and html files/Interleukin-17 and interleukin-22 signaling in psoriasis/7387.html]

MAP3K5


# Protein MAP3K5

|  |  |
| --- | --- |
| URN | urn:agi-llid:4217 |
| Total Entities | 0 |
| Connectivity | 2606 |
| Name | MAP3K5 |
| Description | mitogen-activated protein kinase kinase kinase 5 |
| Notes | Mitogen-activated protein kinase (MAPK) signaling cascades include MAPK or extracellular signal-regulated kinase (ERK), MAPK kinase (MKK or MEK), and MAPK kinase kinase (MAPKKK or MEKK). MAPKK kinase/MEKK phosphorylates and activates its downstream protein kinase, MAPK kinase/MEK, which in turn activates MAPK. The kinases of these signaling cascades are highly conserved, and homologs exist in yeast, Drosophila, and mammalian cells. MAPKKK5 contains 1,374 amino acids with all 11 kinase subdomains. Northern blot analysis shows that MAPKKK5 transcript is abundantly expressed in human heart and pancreas. The MAPKKK5 protein phosphorylates and activates MKK4 (aliases SERK1, MAPKK4) in vitro, and activates c-Jun N-terminal kinase (JNK)/stress-activated protein kinase (SAPK) during transient expression in COS and 293 cells; MAPKKK5 does not activate MAPK/ERK. [provided by RefSeq, Jul 2008] |
| Primary Cell Localization | Cytoplasm |
| Class | Protein kinase |

---

|  |  |
| --- | --- |
| Pathway | Osteoarthritis |
|  | MTHFR Activity Regulation |
|  | Peripheral T-Cell Tolerance |
|  | Leukotriene Effect on Vascular Endothelial Cell Response |
|  | Basophil Activation |
|  | Mast-Cell Activation without Degranulation through TNFSF8 Signaling |
|  | Mast-Cell Activation without Degranulation |
|  | Mast-Cell Activation without Degranulation through IL33/IL1RL1 Signaling |
|  | Mast-Cell Activation without Degranulation through IL1R1 and TLR Signaling |
|  | Macrophage M1 Lineage |
|  | AXL Receptor Inhibits Macrophages and Dendritic Cells Function |
|  | Neutrophil Recruitment and Priming |
|  | Vascular Endothelial Cell Activation by Cytokines |
|  | Vascular Endothelial Cell Activation by Growth Factors |
|  | GFs/TNF -> Ion Channels |
|  | IL1B Expression Targets -> Nociception |
|  | Circadian Clock Genes in Suprachiasmatic Nuclei Neurons |
|  | Apoptosis |
|  | Cardiomyocyte Dysfunction |
|  | Dilated Cardiomyopathy |
|  | Cardiomyocyte Hypertrophy |
|  | Hypertrophic Cardiomyopathy |
|  | Myocardial Remodeling |
|  | Myocardial Infarction |
|  | Proteins Involved in Myocardial Ischemia |
|  | Inflammatory Reaction in Acne Vulgaris |
|  | Keratinocyte Proliferation in Acne Vulgaris |
|  | Mast-Cells Activation in Atopic Dermatitis |
|  | IL17 Signaling in Psoriasis |
|  | Th0-Cell Aberrant Activation in Systemic Lupus Erythematosus |
|  | Vitiligo |
|  | Apoptosis of Melanocyte in Vitiligo |
|  | Podocyte Dysfunction in Diabetic Nephropathy |
|  | Mesangial Cell Dysfunction in Diabetic Nephropathy |
|  | Oxidation of Fatty Acid in non-Alcoholic Fatty Liver Disease |
|  | Nitric Oxide Effects on beta-Cell |
|  | Apoptosis of Thyroid Cell in Hashimoto's Thyroiditis |
|  | Antiphospholipid Antibodies in Endothelial Cell |
|  | Osteoclast Activation in Multiple Myeloma |
|  | Antiphospholipid Antibodies and Endothelial Cell Interaction |
|  | SERPINA1 Associated Liver Damage |
|  | Hepatocellular Carcinoma |
|  | Growth Factor Signaling in Hepatocellular Carcinoma |
|  | Pancreatic Ductal Carcinoma |
|  | Growth Factor Signaling in Pancreatic Neoplasms |
|  | Pancreatic Neuroendocrine Tumors |
|  | TGFBR Signaling in Pancreatic Neoplasms |
|  | IL33 Signaling Related Eosinophilia in Asthma |
|  | CFTR Expression in Epithelial Cells (Class I Mutations) |
|  | Neutrophil Recruitment in Sinovium in Gout |
|  | TNF and IL1B Induce Metalloproteinase Synthesis in Osteoarthritis |
|  | Apoptosis of Chondrocyte |
|  | IL1B Induced Arthralgia |
|  | Synovial Fibroblast Activation in Psoriatic Arthritis |
|  | Synovial Fibroblast Activation by Citokines in Rheumatoid Arthritis |
|  | Apoptosis Block in Synovial Fibroblast |
|  | Autocrine Cytokine/Chemokine Loops in Systemic Scleroderma |
|  | FAS Mediated Apoptosis in Systemic Scleroderma |
|  | Apoptosis Block in Infected Cells |
|  | Diabetes Induced Periodontitis |
|  | IL1 Receptor Antagonist (IL1RN) Deficiency (DIRA) |
|  | TNF Receptor Associated Periodic Syndrome |
|  | IL36 Receptor Antagonist (IL1F5) Deficiency (DITRA) |
|  | ER Stress in Huntington Disease |
|  | Growth Factor Signaling in Neuroblastoma |
|  | Neuroblastoma |
|  | Proteins Involved in Amyotrophic Lateral Sclerosis |
|  | Crystallin alpha B (CRYAB) Suppresses Multiple Sclerosis |
|  | Proteins Involved in Breast Cancer Related to ESR1 Signaling Pathway |
|  | ESR1/ERBB Positive Luminal Breast Cancer |
|  | Breast Cancer |
|  | ESR1 Signaling in Breast Cancer |
|  | Endometrial Cancer |
|  | Endometrioid Endometrial Cancer |
|  | Clear Cell Endometrial Cancer and Papillary Serous Endometrial Cancer |
|  | Endometrioid Ovarian Carcinoma |
|  | Clear Cell Ovarian Carcinoma |
|  | High-Grade Serous Ovarian Carcinoma |
|  | Low-Grade Serous Ovarian Carcinoma |
|  | Ovarian Cancer |
|  | Mucinous Ovarian Carcinoma |
|  | Subepithelial Fibroblasts in Middle Ear Cholesteatoma |
|  | Androgen Receptor/Akt Signaling |
|  | ER Stress in Macular Degeneration |
|  | Steroids Induced Cataract |
|  | MYOC Associated Glaucoma |
|  | Neurotrophic Factor Deprivation in Retinal Ganglion Cell Death |
|  | Cytokines Trigger Otitis Media |
|  | Endothelial Cell Dysfunction in Glomerulonephritis |
|  | Podocyte Dysfunction in Glomerulonephritis |
|  | Mesangial Cell Dysfunction in Glomerulonephritis |
|  | Interstitial Fibroblasts in Pyelonephritis |
|  | Apoptosis of Renal Tubule Epithelial Cell in Pyelonephritis |
|  | Endothelial Cell Dysfunction in Pyelonephritis |
|  | CXCR4 -> ARRB1 Signaling |
|  | AGTR1 -> ARRB1/ARRB2 Signaling |
|  | Sialophorin -> CTNNB/MYC/TP53 Signaling |
|  | TGFB Signaling activation by Blocking of Tumor Suppressors |
|  | Apoptosis Block by Death Receptor Signaling in Cancer |
|  | Apoptosis Evasion in Cancer: Overview |
|  | Effector T-cell Inactivation in Cancer Immune Escape |
|  | IL1R -> NF-kB Signaling |
|  | Elevated Receptors -> Expression Targets in Lymph Node |
|  | NGF -> SMAD3/NF-kB Expression Targets |
|  | NGF -> AP-1/TP53/MYC Expression Targets |
|  | NGF -> FOXO/MYCN/ELK-SRF Expression Targets |
|  | BDNF Expression Targets |
|  | NTF3 Expression Targets |
|  | NTF4 Expression Targets |
|  | NGF -> CREB/CEBPB/MEF2A Expression Targets |
|  | TGFBR -> AP-1 Signaling |
|  | TGFBR -> CREB/ELK-SRF Signaling |
|  | TGFBR -> ATF/GADD/MAX/TP53 Signaling |
|  | TGFBR -> MEF/MYOD/MYOG Signaling |
|  | TNF-alpha/TNFRSF1A Signaling |
|  | JNK/MAPK Signaling |
|  | TGF-beta Signaling |
|  | P38 MAPK/MAPK14 Signaling |
|  | HRAS Signaling |
|  | TGFB3-TGFBR2 Expression Targets |
|  | TGFB2-TGFBR2 Expression Targets |
|  | TGFB1-TGFBR2 Expression Targets |
|  | FASLG Expression Targets |
|  | TNF -> NF-kB Expression Targets |
|  | TNFSF10 Expression Targets |
|  | LTA Expression Targets |
|  | TNFSF14 Expression Targets |
|  | TNF -> AP-1 Expression Targets |
|  | CD72 -> AP-1 Expression Targets |
|  | CD40LG -> NF-kB/ELK/SRF -> CREB/NFATC Expression Targets |
|  | TNFSF13 Expression Targets |
|  | TNFSF13B Expression Targets |
|  | TNF -> ELK-SRF Expression Targets |
|  | TNF -> CREB Expression Targets |
|  | TNF -> TP53/ATF Expression Targets |
|  | EDA Expression Targets |
|  | EctodysplasinR -> NF-kB Signaling |
|  | EctodysplasinR -> AP-1 Signaling |
|  | TNFR -> CREB/ELK-SRF Signaling |
|  | TNFR -> AP-1/ATF/TP53 Signaling |
|  | NGFR -> AP-1/CEBPB/CREB/ELK/SRF/TP53 Signaling |
|  | TNFRSF5/6 -> RB1/E2F Signaling |
|  | NGFR -> NF-kB Signaling |
|  | TNFR -> NF-kB Signaling |
|  | NGFR -> MEF Signaling |
|  | TNFRSF6 -> DDIT3 Signaling |
|  | TNFRSF6 -> HSF1 Signaling |
|  | TNFRSF1A -> AP-1/ATF/TP53 Signaling |
|  | TNFRSF1A -> CREB/ELK-SRF Signaling |
|  | IL1B Expression Targets |
|  | IL1A Expression Targets |
|  | ER Stress (Unfolded Protein Response) |
|  | ROS in Angiotensin Mediated Cardiovascular Remodeling and Hypertrophy |
|  | 1\_Inflammation in keratinocytes in the pathology of acne |
|  | 2\_Interleukin-17 and interleukin-22 signaling in psoriasis |
|  | 1\_TNF and IL1B provoke ECM degradation in osteoarthritis |
|  | 1\_Osteoclast-mediated bone resorption in postmenopausal women |
|  | 2\_Chondrocyte death in osteoarthritis |
|  | 2\_The role of endothelial cells in pyelonephritis |
|  | 3\_The role of interstitial fibroblasts in pyelonephritis |
|  | 3\_Mesangial cell dysfunction in glomerulonephritis |
|  | 2\_Podocyte dysfunction in glomerulonephritis |
|  | 3\_Role of neurotrophic factor deprivation in retinal ganglion cell death |
|  | 2\_MYOC-associated glaucoma |
|  | 3\_Endoplasmic reticulum stress in age-related macular degeneration |
|  | 2\_Cardiomyocyte hypertrophy |
|  | 3\_Cardiomyocyte dysfunction unrelated to cell junctions in ARVD |
|  | 2\_Cigarette smoke and inflammation cause alveolar epithelial cell death in COPD |
|  | 2\_Lipotoxicity in NAFLD |
|  | 2\_HIV - induced depletion of CD4+ T-cells |
|  | 4\_2\_Insulin resistance: inflammation-related insulin resistance |
|  | 4\_3\_Insulin resistance: FFA-related insulin resistance |
|  | 2\_SERPINA1-associated liver damage |
|  | 2\_Neuron and Schwann cell death |
|  | HRAS->RAF->MAPK1/3 |
|  | Interleukin-17 and interleukin-22 signaling in psoriasis |

---

|  |  |
| --- | --- |
| Source | curated |

---

|  |  |
| --- | --- |
| MedScan ID | 4217 |
|  | 365057 |
|  | 293015 |

---

|  |  |
| --- | --- |
| LocusLink ID | 4217 |
|  | 26408 |
|  | 365057 |
|  | 293015 |
|  | 17273 |
|  | 320994 |
|  | 537380 |
|  | 555703 |
|  | 336299 |
|  | 373122 |
|  | 555773 |
|  | 326408 |

---

|  |  |
| --- | --- |
| Alias | MAPKKK5 |
|  | MAPK/ ERK kinase kinase V |
|  | 7420452D20 |
|  | mitogen-activated protein kinase kinase kinase V |
|  | MGC141518 |
|  | RP3-325F22.4 |
|  | MAP kinase kinase kinase 5 |
|  | ASK1 |
|  | MEK kinase 5 |
|  | OTTMUSP00000040576 |
|  | MGC141519 |
|  | OTTHUMP00000017275 |
|  | LOC270672 |
|  | Map3k5 |
|  | MAP/ERK kinase kinase 5 |
|  | mitogen activated protein kinase kinase kinase 5 |
|  | RGD1306565 |
|  | apoptosis signal regulating kinase |
|  | MAP/ERK kinase kinase V |
|  | MAPK/ ERK kinase kinase 5 |
|  | 7420452D20Rik |
|  | apoptosis signaling-regulating kinase 1 |
|  | Apoptosis signal- regulating kinase 1 |
|  | MEKK5 |
|  | ASK I |
|  | Apoptosis signal- regulating kinase I |
|  | RIKEN cDNA 7420452D20 gene |
|  | MEK kinase V |
|  | ASK-1 |
|  | MAPK/ERK kinase kinase 5 |
|  | MEKK 5 |
|  | apoptosis signal-regulating kinase 1 |
|  | mitogen-activated protein kinase kinase kinase 5 |
|  | ASK |
|  | apoptosis signal regulating kinase 1 |
|  | hypothetical protein LOC270672 |
|  | similar to ASK I |
|  | similar to ASK1 |
|  | Rtc I |
|  | scaffold |
|  | Resistance to Tongue Carcinomas QTL I |
|  | fj62c10 |
|  | chromosome |
|  | LOC555773 |
|  | Map3k5\_predicted |
|  | Resistance to Tongue Carcinomas QTL 1 |
|  | similar to mitogen-activated protein kinase kinase kinase 5 |
|  | Ttr1 |
|  | wu:fj62c10 |
|  | Rtc1 |

---

|  |  |
| --- | --- |
| EC Number | 2.7.11.25 |
|  | 2.7.1.- |

---

|  |  |
| --- | --- |
| GO ID | 0005524 |
|  | 0004709 |
|  | 0000287 |
|  | 0019904 |
|  | 0042803 |
|  | 0004672 |
|  | 0019901 |
|  | 0019903 |
|  | 0004674 |
|  | 0007254 |
|  | 0000165 |
|  | 0007257 |
|  | 0000186 |
|  | 0097190 |
|  | 0034198 |
|  | 0070301 |
|  | 1902170 |
|  | 0071356 |
|  | 0072577 |
|  | 0045087 |
|  | 0070059 |
|  | 0008631 |
|  | 0038066 |
|  | 0046330 |
|  | 0043507 |
|  | 0043065 |
|  | 0010666 |
|  | 0043280 |
|  | 0045663 |
|  | 1901216 |
|  | 1900745 |
|  | 0045893 |
|  | 1904707 |
|  | 0097300 |
|  | 0006468 |
|  | 0034976 |
|  | 0002931 |
|  | 0051403 |
|  | 0016032 |
|  | 0042060 |
|  | 1990604 |
|  | 0005829 |
|  | 0009897 |
|  | 1902911 |
|  | 0032991 |
|  | 0016301 |
|  | 0046872 |
|  | 0000166 |
|  | 0016740 |
|  | 0000187 |
|  | 0006915 |
|  | 0002376 |
|  | 0016310 |
|  | 0001934 |
|  | 0010941 |
|  | 0005737 |
|  | 0005783 |
|  | 0008656 |
|  | 0006919 |
|  | 0016772 |
|  | 0005622 |
|  | 0019048 |
|  | 0006950 |
|  | 0010421 |
|  | 0008624 |
|  | 0044419 |
|  | 0010952 |
|  | 0043281 |
|  | 0005515 |
|  | 0042802 |
|  | 0005575 |
|  | 0004713 |
|  | 0004706 |
|  | 0031434 |
|  | 0046982 |
|  | 0007256 |
|  | 0046777 |
|  | 0051291 |
|  | 0005938 |
|  | 0005634 |

---

|  |  |
| --- | --- |
| KEGG ID | hsa:4217 |
|  | mmu:26408 |
|  | rno:365057 |

---

|  |  |
| --- | --- |
| MedLine Reference | 12050113 |
|  | 12869527 |
|  | 15629441 |
|  | 16006035 |
|  | 16316999 |
|  | 16648474 |
|  | 16762504 |
|  | 16891268 |
|  | 17015265 |
|  | 10349636 |
|  | 11042159 |
|  | 11076861 |
|  | 11217851 |
|  | 11266364 |
|  | 11959862 |
|  | 12215209 |
|  | 12391142 |
|  | 12466851 |
|  | 12477932 |
|  | 12724324 |
|  | 14551246 |
|  | 14665690 |
|  | 14668338 |
|  | 15467832 |
|  | 15767678 |
|  | 15864310 |
|  | 15938714 |
|  | 15953587 |
|  | 15976330 |
|  | 16109778 |
|  | 16141072 |
|  | 16141073 |
|  | 16400028 |
|  | 16449798 |
|  | 16731828 |
|  | 9367868 |
|  | 10411906 |
|  | 10523862 |
|  | 10734135 |
|  | 10921914 |
|  | 11003656 |
|  | 11090355 |
|  | 11096076 |
|  | 11154276 |
|  | 11278289 |
|  | 11336675 |
|  | 11356842 |
|  | 11416155 |
|  | 11427728 |
|  | 11493600 |
|  | 11689443 |
|  | 11920685 |
|  | 12077134 |
|  | 12089063 |
|  | 12189133 |
|  | 12244106 |
|  | 12372597 |
|  | 12473108 |
|  | 12482984 |
|  | 12543931 |
|  | 12556535 |
|  | 12566458 |
|  | 12697749 |
|  | 12813029 |
|  | 12819028 |
|  | 12912994 |
|  | 12968034 |
|  | 14688258 |
|  | 14761963 |
|  | 15023544 |
|  | 15210709 |
|  | 15218033 |
|  | 15246877 |
|  | 15310755 |
|  | 15328343 |
|  | 15670787 |
|  | 15701637 |
|  | 15755722 |
|  | 15782121 |
|  | 15843568 |
|  | 15864780 |
|  | 15866172 |
|  | 15925322 |
|  | 16476732 |
|  | 16512785 |
|  | 16515547 |
|  | 16532036 |
|  | 16636664 |
|  | 16644673 |
|  | 8940179 |
|  | 8974401 |
|  | 9465908 |
|  | 9564042 |
|  | 9651337 |
|  | 9743501 |
|  | 9774977 |
|  | 9875215 |
|  | 10917738 |
|  | 12618376 |
|  | 12641569 |
|  | 11298454 |
|  | 14574404 |
|  | 15094778 |
|  | 15489334 |
|  | 17210579 |
|  | 17220297 |
|  | 17244475 |
|  | 17325029 |
|  | 17331470 |
|  | 17389591 |
|  | 17456047 |
|  | 17543279 |
|  | 17068291 |
|  | 17219248 |
|  | 17389227 |
|  | 17522316 |
|  | 17602894 |
|  | 17646574 |

---

|  |  |
| --- | --- |
| Organism | Homo sapiens {Organism urn:agi-taxid:9606} |
|  | Mus musculus {Organism urn:agi-taxid:10090} |
|  | Rattus norvegicus {Organism urn:agi-taxid:10116} |
|  | Homo sapiens |
|  | Mus musculus |
|  | Rattus norvegicus |
|  | Bos taurus |
|  | Danio rerio |
|  | Human |

---

|  |  |
| --- | --- |
| Mouse chromosome position | 10 |
|  | 10 A3 |

---

|  |  |
| --- | --- |
| OMIM ID | 602448 |
|  | OMIM:602448 |
|  | 61399 |

---

|  |  |
| --- | --- |
| Rat chromosome position | 1p12 |

---

|  |  |
| --- | --- |
| Hugo ID | 6857 |
|  | HGNC:6857 |

---

|  |  |
| --- | --- |
| Human chromosome position | 6q23.3 |
|  | 6q22.33 |

---

|  |  |
| --- | --- |
| Swiss-Prot Accession | Q99683 |
|  | Q99683.1 |
|  | O35099 |
|  | O35099.3 |
|  | A6NIA0 |
|  | B4DGB2 |
|  | Q5THN3 |
|  | Q99461 |
|  | Q14AY5 |
|  | Q59GL6 |
|  | Q14AY4 |
|  | Q8CC79 |
|  | M3K5\_HUMAN |
|  | Q14AY5\_MOUSE |

---

|  |  |
| --- | --- |
| PIR ID | JC5778 |

---

|  |  |
| --- | --- |
| GenBank ID | NC\_000006 |
|  | XM\_017010875 |
|  | XP\_016866364 |
|  | XM\_017010872 |
|  | XP\_016866361 |
|  | XM\_017010873 |
|  | XP\_016866362 |
|  | NM\_005923 |
|  | NP\_005914 |
|  | XM\_017010877 |
|  | XP\_016866366 |
|  | XM\_017010874 |
|  | XP\_016866363 |
|  | XM\_017010871 |
|  | XP\_016866360 |
|  | XM\_017010870 |
|  | XP\_016866359 |
|  | XM\_017010876 |
|  | XP\_016866365 |
|  | XM\_011535839 |
|  | XP\_011534141 |
|  | NG\_011965 |
|  | AL024508 |
|  | AL121933 |
|  | AY605043 |
|  | AAT52164 |
|  | CH471051 |
|  | EAW47942 |
|  | KT584492 |
|  | AB209093 |
|  | BAD92330 |
|  | AK293953 |
|  | BAG57328 |
|  | AK294507 |
|  | BAG57723 |
|  | AK310942 |
|  | BC054503 |
|  | AAH54503 |
|  | BC088829 |
|  | AAH88829 |
|  | D84476 |
|  | BAA12684 |
|  | U67156 |
|  | AAC50894 |
|  | Q99683 |
|  | NC\_000076 |
|  | XR\_001779529 |
|  | XM\_006512739 |
|  | XP\_006512802 |
|  | NM\_008580 |
|  | NP\_032606 |
|  | AC158620 |
|  | AC158621 |
|  | AC168883 |
|  | AC171277 |
|  | CH466562 |
|  | EDL03445 |
|  | AB006787 |
|  | BAA23648 |
|  | AK033726 |
|  | BAC28449 |
|  | AK078668 |
|  | BC116627 |
|  | AAI16628 |
|  | BC116628 |
|  | AAI16629 |
|  | BC133697 |
|  | AAI33698 |
|  | O35099 |
|  | NC\_005100 |
|  | NM\_001277694 |
|  | NP\_001264623 |
|  | AC\_000069 |
|  | AABR07000495 |
|  | AABR07000496 |
|  | AABR07000497 |
|  | AABR07000498 |
|  | AABR07000499 |
|  | AABR07000500 |
|  | AAHX01001718 |
|  | AAHX01001719 |
|  | AAHX01001720 |
|  | AAHX01001721 |
|  | AAHX01001722 |
|  | AAHX01001723 |
|  | AAHX01001724 |
|  | AAHX01001725 |
|  | AAHX01001726 |
|  | AAHX01001727 |
|  | AAHX01001728 |
|  | AAHX01001729 |
|  | AAHX01001730 |
|  | AAHX01001731 |
|  | AAHX01001732 |
|  | AAHX01001733 |
|  | AAHX01001734 |
|  | AAHX01001735 |
|  | AAHX01001736 |
|  | CH473994 |
|  | EDL93792 |
|  | XM\_011535838 |
|  | XP\_011534140 |
|  | XM\_011535837 |
|  | XP\_011534139 |
|  | NC\_018917 |
|  | AMYH02014559 |
|  | AMYH02014560 |
|  | AMYH02014561 |
|  | AC\_000032 |
|  | AAHY01083852 |
|  | AAHY01083853 |
|  | AAHY01083854 |
|  | AAHY01083855 |
|  | AAHY01083856 |
|  | AAHY01083857 |
|  | AAHY01083858 |
|  | AAHY01083859 |
|  | AAHY01083860 |
|  | AAHY01083861 |
|  | AAHY01083862 |
|  | AAHY01083863 |
|  | AAHY01083864 |
|  | AAHY01083865 |
|  | AAHY01083866 |
|  | AAHY01083867 |
|  | AAHY01083868 |
|  | AAHY01083869 |
|  | AAHY01083870 |
|  | AAHY01083871 |
|  | XM\_008758622 |
|  | XP\_008756844 |
|  | XM\_005266990 |
|  | XP\_005267047 |
|  | XM\_006715482 |
|  | XP\_006715545 |
|  | XM\_005266991 |
|  | XP\_005267048 |
|  | XM\_005266992 |
|  | XP\_005267049 |
|  | AC\_000138 |
|  | ABBA01025825 |
|  | ABBA01025826 |
|  | AABR06000786 |
|  | AABR06000787 |
|  | AABR06000788 |
|  | AABR06000789 |
|  | AABR06000790 |
|  | AABR06000791 |
|  | AABR06000792 |
|  | AABR06000793 |
|  | AABR06000794 |
|  | AABR06000795 |
|  | CAI20176 |
|  | CAI15470 |
|  | XR\_145729 |
|  | XR\_146724 |
|  | NT\_025741 |
|  | NW\_923184 |
|  | NW\_001838990 |
|  | AC\_000049 |
|  | Q59GL6 |
|  | NT\_039492 |
|  | NW\_001030408 |
|  | Q14AY4 |
|  | Q14AY5 |
|  | Q8CC79 |
|  | NW\_032785 |
|  | XP\_001006040 |
|  | XM\_001006040 |
|  | XM\_913629 |
|  | Q5THN3 |
|  | 109730385 |
|  | 109733254 |
|  | 123782812 |
|  | 123792789 |
|  | 26329421 |
|  | 68533198 |
|  | 81877629 |
|  | 82654957 |
|  | 119568327 |
|  | 1679668 |
|  | 32452016 |
|  | 55957178 |
|  | 56202652 |
|  | 57033166 |
|  | 62087766 |
|  | 66391167 |
|  | 6685617 |
|  | 74741299 |
|  | NT\_086697 |
|  | NW\_047545 |
|  | XM\_218780 |
|  | XP\_218780 |
|  | XP\_918722 |
|  | NC\_007307 |
|  | NW\_001495597 |
|  | XM\_617542 |
|  | XP\_617542 |
|  | AW282104 |
|  | AW305675 |
|  | 56208086 |
|  | 56311422 |
|  | AI497484 |
|  | AL922223 |
|  | BX248523 |
|  | BX545848 |
|  | CAI21268 |
|  | CAI29402 |
|  | 123913850 |
|  | 82196411 |
|  | DU645016 |
|  | NC\_007131 |
|  | NW\_001512062 |
|  | Q5PNR2 |
|  | Q5RHC1 |
|  | XM\_678295 |
|  | XP\_683387 |
|  | 56311423 |
|  | CAI29403 |
|  | 126631249 |
|  | 148671498 |
|  | 5174547 |
|  | 57033165 |
|  | 1805500 |
|  | 51465807 |
|  | 1679667 |
|  | 37551196 |
|  | 1805499 |
|  | 21536459 |
|  | 6678795 |
|  | 6678796 |
|  | 2653433 |
|  | 2653434 |
|  | 26098027 |
|  | NT\_039491 |
|  | 26329420 |
|  | 34193967 |
|  | 6685611 |
|  | 34852815 |
|  | 51765492 |
|  | 34852812 |
|  | 34852811 |

---

|  |  |
| --- | --- |
| Swiss-Prot ID | M3K5\_HUMAN |
|  | M3K5\_MOUSE |
|  | Q14AY5\_MOUSE |

---

|  |  |
| --- | --- |
| Cell Localization | Cytoplasm |
|  | Endoplasmic reticulum |

---

|  |  |
| --- | --- |
| Ensembl ID | ENSG00000197442 |
|  | ENSP00000351908.4 |
|  | ENST00000359015.5 |
|  | ENSMUSG00000071369 |
|  | ENSMUSP00000093485.3 |
|  | ENSMUST00000095806.9 |
|  | ENSRNOG00000031700 |
|  | ENSRNOP00000051338.5 |
|  | ENSRNOT00000051496.6 |
|  | ENSP00000351908 |
|  | ENST00000359015 |
|  | ENSMUSP00000093485 |
|  | ENSMUST00000095806 |
|  | ENSRNOP00000051338 |
|  | ENSRNOT00000051496 |

---

|  |  |
| --- | --- |
| MGI ID | MGI:1346876 |
|  | 1346876 |
|  | 1101761 |
|  | 2445207 |

---

|  |  |
| --- | --- |
| RGD ID | 1306565 |
|  | 1306431 |
|  | 61399 |

---

|  |  |
| --- | --- |
| Unigene ID | Hs.186486 |
|  | Mm.6595 |
|  | Rn.232064 |
|  | Rn.108921 |
|  | Hs.151988 |
|  | Bt.31917 |
|  | Dr.122363 |
|  | Dr.16458 |
|  | Dr.27055 |

---

|  |  |
| --- | --- |
| Homologene ID | 38114 |

---

|  |  |
| --- | --- |
| Shape | Sickle-vertex |

---

|  |  |
| --- | --- |
| IPI ID | IPI00412433 |
|  | IPI00126589 |
|  | IPI00890883 |
|  | IPI00479216 |
|  | IPI00748581 |
|  | IPI00894869 |
|  | IPI00895076 |
|  | IPI00894925 |

---

|  |  |
| --- | --- |
| Chromosome position | 6q22.33 |
|  | 1p12 |
|  | 10 A3 |

---

|  |  |
| --- | --- |
| Microarray ID | 99855\_at |
|  | 203837\_at |
|  | A\_51\_P300986 |
|  | ab006787\_at |
|  | U67156 |
|  | MA206 |
|  | U67156\_at |
|  | A\_23\_P134125 |
|  | A\_14\_P104796 |
|  | 1327\_s\_at |
|  | rc\_aa057432\_s\_at |
|  | A\_14\_P126311 |
|  | 203836\_s\_at |
|  | OH8272 |
|  | A\_14\_P112751 |
|  | A\_14\_P118658 |
|  | A\_14\_P110978 |
|  | CA078 |
|  | 1421340\_at |

---

|  |  |
| --- | --- |
| Homo sapiens Chromosome position | 6q22.33 |

---

|  |  |
| --- | --- |
| Ensembl Transcript ID | ENST00000359015 |

---

|  |  |
| --- | --- |
| ENSEMBL | ENSG00000197442 |

---

|  |  |
| --- | --- |
| FunctionalClass | MAP kinase kinase kinase |
|  | caspase activator |
|  | identical protein binding |
|  | protein binding |
|  | ATP binding |
|  | kinase |
|  | magnesium ion binding |
|  | metal ion binding |
|  | nucleotide binding |
|  | protein kinase |
|  | protein serine/threonine kinase |
|  | transferase |
|  | protein self binding |
|  | protein homodimerization |
|  | protein-tyrosine kinase |

---

|  |  |
| --- | --- |
| Hugo Symbol | MAP3K5 |

---

|  |  |
| --- | --- |
| Bos taurus Chromosome position | 9 |

---

|  |  |
| --- | --- |
| Mus musculus Chromosome position | 10 A3 |

---

|  |  |
| --- | --- |
| KEGG pathway | MAPK signaling pathway |

---

|  |  |
| --- | --- |
| Rattus norvegicus Chromosome position | 1p12 |

---

|  |  |
| --- | --- |
| Ensembl Gene ID | ENSG00000197442 |

---

|  |  |
| --- | --- |
| ProteinAtlas NormalTissue | Tissue: adrenal gland; Cell type: glandular cells; Level: Moderate; Expression type: Staining; Reliability: Uncertain |
|  | Tissue: appendix; Cell type: glandular cells; Level: Negative; Expression type: Staining; Reliability: Uncertain |
|  | Tissue: appendix; Cell type: lymphoid tissue; Level: Weak; Expression type: Staining; Reliability: Uncertain |
|  | Tissue: bone marrow; Cell type: hematopoietic cells; Level: Weak; Expression type: Staining; Reliability: Uncertain |
|  | Tissue: breast; Cell type: glandular cells; Level: Negative; Expression type: Staining; Reliability: Uncertain |
|  | Tissue: bronchus; Cell type: respiratory epithelial cells; Level: Moderate; Expression type: Staining; Reliability: Uncertain |
|  | Tissue: cerebellum; Cell type: cells in granular layer; Level: Negative; Expression type: Staining; Reliability: Uncertain |
|  | Tissue: cerebellum; Cell type: cells in molecular layer; Level: Negative; Expression type: Staining; Reliability: Uncertain |
|  | Tissue: cerebellum; Cell type: Purkinje cells; Level: Negative; Expression type: Staining; Reliability: Uncertain |
|  | Tissue: cerebral cortex; Cell type: glial cells; Level: Negative; Expression type: Staining; Reliability: Uncertain |
|  | Tissue: cerebral cortex; Cell type: neuronal cells; Level: Negative; Expression type: Staining; Reliability: Uncertain |
|  | Tissue: cervix, uterine; Cell type: glandular cells; Level: Negative; Expression type: Staining; Reliability: Uncertain |
|  | Tissue: cervix, uterine; Cell type: squamous epithelial cells; Level: Negative; Expression type: Staining; Reliability: Uncertain |
|  | Tissue: colon; Cell type: glandular cells; Level: Weak; Expression type: Staining; Reliability: Uncertain |
|  | Tissue: duodenum; Cell type: glandular cells; Level: Negative; Expression type: Staining; Reliability: Uncertain |
|  | Tissue: epididymis; Cell type: glandular cells; Level: Moderate; Expression type: Staining; Reliability: Uncertain |
|  | Tissue: esophagus; Cell type: squamous epithelial cells; Level: Negative; Expression type: Staining; Reliability: Uncertain |
|  | Tissue: fallopian tube; Cell type: glandular cells; Level: Negative; Expression type: Staining; Reliability: Uncertain |
|  | Tissue: gall bladder; Cell type: glandular cells; Level: Weak; Expression type: Staining; Reliability: Uncertain |
|  | Tissue: heart muscle; Cell type: myocytes; Level: Negative; Expression type: Staining; Reliability: Uncertain |
|  | Tissue: hippocampus; Cell type: glial cells; Level: Negative; Expression type: Staining; Reliability: Uncertain |
|  | Tissue: hippocampus; Cell type: neuronal cells; Level: Negative; Expression type: Staining; Reliability: Uncertain |
|  | Tissue: kidney; Cell type: cells in glomeruli; Level: Negative; Expression type: Staining; Reliability: Uncertain |
|  | Tissue: kidney; Cell type: cells in tubules; Level: Weak; Expression type: Staining; Reliability: Uncertain |
|  | Tissue: lateral ventricle; Cell type: glial cells; Level: Negative; Expression type: Staining; Reliability: Uncertain |
|  | Tissue: lateral ventricle; Cell type: neuronal cells; Level: Negative; Expression type: Staining; Reliability: Uncertain |
|  | Tissue: liver; Cell type: bile duct cells; Level: Negative; Expression type: Staining; Reliability: Uncertain |
|  | Tissue: liver; Cell type: hepatocytes; Level: Negative; Expression type: Staining; Reliability: Uncertain |
|  | Tissue: lung; Cell type: macrophages; Level: Moderate; Expression type: Staining; Reliability: Uncertain |
|  | Tissue: lung; Cell type: pneumocytes; Level: Negative; Expression type: Staining; Reliability: Uncertain |
|  | Tissue: lymph node; Cell type: germinal center cells; Level: Negative; Expression type: Staining; Reliability: Uncertain |
|  | Tissue: lymph node; Cell type: non-germinal center cells; Level: Weak; Expression type: Staining; Reliability: Uncertain |
|  | Tissue: nasopharynx; Cell type: respiratory epithelial cells; Level: Strong; Expression type: Staining; Reliability: Uncertain |
|  | Tissue: oral mucosa; Cell type: squamous epithelial cells; Level: Negative; Expression type: Staining; Reliability: Uncertain |
|  | Tissue: ovary; Cell type: follicle cells; Level: Negative; Expression type: Staining; Reliability: Uncertain |
|  | Tissue: ovary; Cell type: ovarian stroma cells; Level: Weak; Expression type: Staining; Reliability: Uncertain |
|  | Tissue: pancreas; Cell type: exocrine glandular cells; Level: Weak; Expression type: Staining; Reliability: Uncertain |
|  | Tissue: pancreas; Cell type: islets of Langerhans; Level: Negative; Expression type: Staining; Reliability: Uncertain |
|  | Tissue: parathyroid gland; Cell type: glandular cells; Level: Negative; Expression type: Staining; Reliability: Uncertain |
|  | Tissue: placenta; Cell type: decidual cells; Level: Negative; Expression type: Staining; Reliability: Uncertain |
|  | Tissue: placenta; Cell type: trophoblastic cells; Level: Negative; Expression type: Staining; Reliability: Uncertain |
|  | Tissue: prostate; Cell type: glandular cells; Level: Moderate; Expression type: Staining; Reliability: Uncertain |
|  | Tissue: rectum; Cell type: glandular cells; Level: Weak; Expression type: Staining; Reliability: Uncertain |
|  | Tissue: salivary gland; Cell type: glandular cells; Level: Negative; Expression type: Staining; Reliability: Uncertain |
|  | Tissue: seminal vesicle; Cell type: glandular cells; Level: Negative; Expression type: Staining; Reliability: Uncertain |
|  | Tissue: skeletal muscle; Cell type: myocytes; Level: Negative; Expression type: Staining; Reliability: Uncertain |
|  | Tissue: skin; Cell type: epidermal cells; Level: Negative; Expression type: Staining; Reliability: Uncertain |
|  | Tissue: small intestine; Cell type: glandular cells; Level: Negative; Expression type: Staining; Reliability: Uncertain |
|  | Tissue: smooth muscle; Cell type: smooth muscle cells; Level: Negative; Expression type: Staining; Reliability: Uncertain |
|  | Tissue: spleen; Cell type: cells in red pulp; Level: Negative; Expression type: Staining; Reliability: Uncertain |
|  | Tissue: spleen; Cell type: cells in white pulp; Level: Weak; Expression type: Staining; Reliability: Uncertain |
|  | Tissue: stomach, lower; Cell type: glandular cells; Level: Weak; Expression type: Staining; Reliability: Uncertain |
|  | Tissue: stomach, upper; Cell type: glandular cells; Level: Moderate; Expression type: Staining; Reliability: Uncertain |
|  | Tissue: testis; Cell type: cells in seminiferus ducts; Level: Moderate; Expression type: Staining; Reliability: Uncertain |
|  | Tissue: testis; Cell type: Leydig cells; Level: Moderate; Expression type: Staining; Reliability: Uncertain |
|  | Tissue: thyroid gland; Cell type: glandular cells; Level: Negative; Expression type: Staining; Reliability: Uncertain |
|  | Tissue: tonsil; Cell type: germinal center cells; Level: Negative; Expression type: Staining; Reliability: Uncertain |
|  | Tissue: tonsil; Cell type: non-germinal center cells; Level: Weak; Expression type: Staining; Reliability: Uncertain |
|  | Tissue: tonsil; Cell type: squamous epithelial cells; Level: Weak; Expression type: Staining; Reliability: Uncertain |
|  | Tissue: urinary bladder; Cell type: urothelial cells; Level: Strong; Expression type: Staining; Reliability: Uncertain |
|  | Tissue: uterus, post-menopause; Cell type: cells in endometrial stroma; Level: Weak; Expression type: Staining; Reliability: Uncertain |
|  | Tissue: uterus, post-menopause; Cell type: glandular cells; Level: Weak; Expression type: Staining; Reliability: Uncertain |
|  | Tissue: uterus, pre-menopause; Cell type: cells in endometrial stroma; Level: Negative; Expression type: Staining; Reliability: Uncertain |
|  | Tissue: uterus, pre-menopause; Cell type: glandular cells; Level: Moderate; Expression type: Staining; Reliability: Uncertain |
|  | Tissue: vagina; Cell type: squamous epithelial cells; Level: Negative; Expression type: Staining; Reliability: Uncertain |
|  | Tissue: vulva/anal skin; Cell type: epidermal cells; Level: Negative; Expression type: Staining; Reliability: Uncertain |

---

|  |  |
| --- | --- |
| Danio rerio Chromosome position | LG 25 |

---
